# Flow diverter stent for treatment of cerebral aneurysms: A report of 130 patients with 134 aneurysms

**DOI:** 10.1016/j.heliyon.2020.e03356

**Published:** 2020-02-04

**Authors:** Nguyen Thai Binh, Vu Dang Luu, Pham Minh Thong, Nguyen Ngoc Cuong, Nguyen Quang Anh, Tran Anh Tuan, Le Tuan Linh, Nguyen Tat Thien, Md Jamal Uddin, Thien Chu Dinh, Dinh-Toi Chu

**Affiliations:** a Radiology Department, Hanoi Medical University Hospital, Hanoi, Viet Nam; b Hanoi Medical University, Hanoi, Viet Nam; c Bach Mai Hospital, Hanoi, Viet Nam; d College of Pharmacy, Ewha Womans University, Seoul, South Korea; e Institute for Research and Development, Duy Tan University, 03 Quang Trung, Danang, Viet Nam; f Hanoi National University of Education, Hanoi, Viet Nam; g School of Odonto Stomatology, Hanoi Medical University, Hanoi, Viet Nam

**Keywords:** Health sciences, Neurology, Surgery, Alternative medicine, Emergency medicine, Cavernous segments, Flow diverter stent, Intracranial aneurysms, Para-ophthalmic segments, Aneurysms

## Abstract

**Background:**

This study aims to report our experience with cerebral aneurysms, which may improve in the treatment with the flow-diverter stent and follow up.

**Methods:**

This study was conducted in a consecutive series of 130 patients. 134 procedures were performed for treating these patients in Hanoi Medical University Hospital and Bach Mai Hospital from January 2012 to April 2017. 143 flow diverter stents (Pipeline, FRED and SILK) were used. Aneurysm morphology, stent patency and cerebral parenchyma before and after intervention were analyzed on images of digital subtraction angiography (DSA), computed tomography (CT) and magnetic resonance (MR). The follow-up data after 3–6 months and 12 months were recorded.

**Results:**

In 130 patients (31 men, 99 women), aneurysms of internal carotid artery were mostly common (92.6%), especially in cavernous (35.1%) and in para-ophthalmic (40.3%) segments. 83 cases (61.9%) had wide-neck aneurysms, and 16 cases (11.9%) had multiple aneurysms, and only 5 cases (3.7%) had blister-liked aneurysms. Endovascular treatment was successfully performed at rate of 94.8%. In 3 patients, the stent could not be delivered. Mortality and morbidity rates were 1.5% and 3.7%, respectively. MRI and MSCT follow-up at 3 months showed complete or incomplete occlusions of aneurysms was 7.4% or 17.5%, respectively. 3 patients experienced a thromboembolic event (4.3%).

**Conclusions:**

Intracranial aneurysms of cavernous and para-ophthalmic segments of internal carotid artery are mostly common with wide-neck and multi aneurysms. Deployment of flow diverter stent is safe and effective with high rate of successful and low procedural complications.

## 1 Introduction

Cerebral aneurysm is a relatively common disease which may occur from 2.3 to 5% of the population seemed to increase fast in Vietnam in some recent years [1]. This health issue causes high mortality risk (40–45%) for patients if the aneurysm is ruptured [2]. The risk of rupture depends on many factors including patient related (arterial hypertension, smoking, alcohol abuse, age, geographical location…) and aneurysm related (size, location, shape etc.) [[3], [4]]. In addition, challenging cases of giant, blister-like, fusiform aneurysms… lead to difficult operation of treatment for both clipping and even coiling intervention with high complications as well as recurrence rate. Interventional therapy, with its break through in technology development of treatment in the last decade, already helped solve this problem by the appearance of flow-diverter. This kind of stent now is one of the most effective alternative treatment that has been widely used for treating cerebral aneurysm around the world.

Kallmes et al., have conducted a clinical trial using stent Pipeline NED in rabbits and reported that the stent reduces the flow resulting in aneurysm thrombosis but preserves the parent artery and lateral branches. The next generation of flow diverter stents covers about 30–35% of the wall area, and can be redeployed if necessary. Some previous studies in the world have shown that this method has a very high success rate (93–95%) with low complication rate (2.3–5.6%) [5, 6, 7].

In Vietnam, flow diverter stents were first introduced in 2009 in the Bach Mai Radiology Center. In this study, we have summarized the results of this method from from January 2012 to April 2017 with 130 patients treated at Bach Mai University hospital and Hanoi Medical University hospital with two objectives: 1) to describe the characteristics of cerebral aneurysm treated with flow diverter stents, and 2) to evaluate the early results of stenting method.

## 2 Patients and methods

### 2.1 Patient selection

130 patients were selected in this study with the inclusion criteria:1) older than 18 years, 2) having complicated un-ruptured or ruptured aneurysms (They had been treated through acute stage), and 3) being treated with flow diverter stent in two studied hospitals from January 2012 to April 2017.

The exclusion criteria:1) patients should not take the dual anti-coagulant medication exactly according to the regimen, and 2) Patient's refusal.

All procedures performed in studies involving human participants were in accordance with the ethical standards of the institutional and/or national research committee and with the 1964 Helsinki declaration and its later amendments or comparable ethical standards. This research was approved by the scientific ethics committee of Hanoi Medical University Hospital.

### 2.2 Methods

This is a retrospective and prospective study. Research facilities in studied hospitals include Digital subtraction angiography system Phillip AlluraXper, multi slides computed tomography (64 slides or more), 1.5T magnetic resonance imaging, and flow diverter stents (Pipeline (Covidien), FRED (Microvention) or Silk (Balt)).

### 2.3 Procedure and follow up

- Imaging characteristics by CT Angio, MR Angio or DSA angiogram of the patients were recorded: Morphology, diameters of the aneurysms, changes of degree of angiographic filling and contrast stasis before and right after stenting (according to the O'Kelly-Marotta (OKM) grading scale)
- 3–5 days before procedure, the patients were given dual anti-platelet medications (clopidogrel 75 mg/day and aspirin 100 mg/day). Heparine bolus (intra arterial) 2500 UI was given to patients before procedure and maintained a dose of 500–1000 UI/h intra procedure. Patients were given dual anti-platelet therapy for 6 months and maintained aspirin 100 mg/day for the next 6 months.
- The 2^nd^ stent were added if the 1^st^ stent were not fully covered the neck of aneurysm or displacement one tip into the aneurysm. Coils additional were use if need if the aneurysm were ruptured or have high risk of rupture (large or giant aneurysm with irregular shape).
- Technical success criteria were the stent, which was opened >75% diameter of the parent artery and covered the neck of aneurysm.
- Early and late complications, imaging of cerebral arteries, parenchyma and aneurysms were analyzed on CT angio and MR angio after 3 months, and 6–12 months of intervention.

## 3 Results

The study included 134 procedures of 130 patients (two patients had aneurysms of bilateral internal carotid arteries and one cases of vertebral aneurysm was treated in 3 procedures). The female/male ratio of the study groups was 3.19, and the median age was 50, ranged from 22- 76 years old.

### 3.1 Imaging of aneurysms by DSA

**Figure 1:**
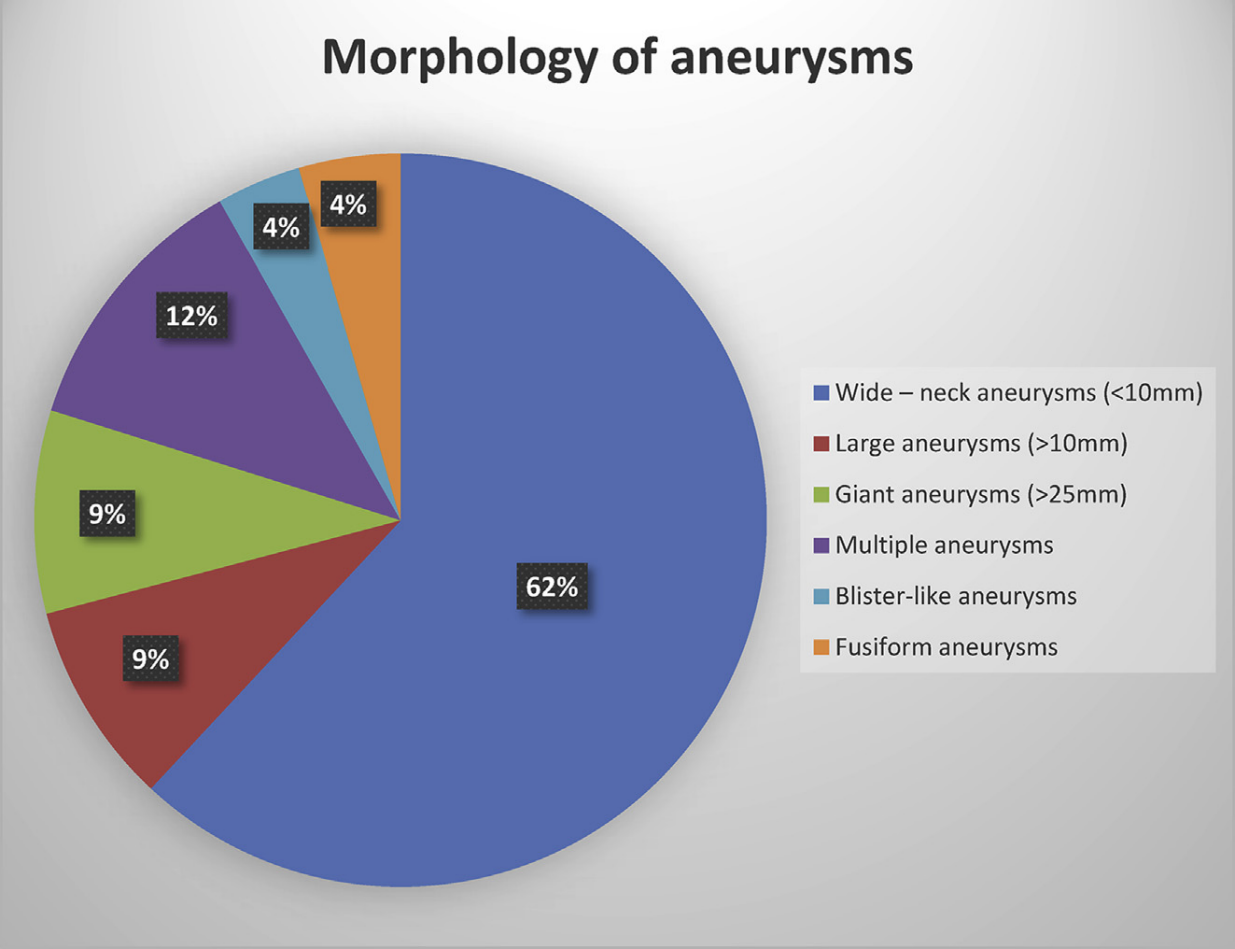
Morphology of aneurysms.
- Size: Diameter average: 6.6 mm (±5.3), with a minimum diameter of 1.7 mm, and maximum diameter of 28 mm; aneurysm high average: 7.2 mm (±6.3), of which the smallest was 2 mm, and the biggest was 39 mm. The neck diameter average was about 5.1 mm. The dome/neck ratio was 1.4.
- Localization: aneurysms commonly located in the cavernous segment and the para-ophthalmic segment or multi locations with the rate of 35.1%, 40.3%, or 11.9%, respectively (Table 1).

- Morphology: Aneurysm morphology showed in Figure 1 with wide-neck, large (>10mm) giant (≥25mm), multiple, blister-like and fusiform aneurysms in 62%, 9%, 9%, 12%, 4% and 4%, respectively.

**Table 1:** Localization of aneurysms.

Localization		N	%
Carotid artery	Cavernous segment	47	35.1
	Para-ophthalmic segment	54	40.3
	Posterior communicating segment	3	2.2
	Anterior Choroid segment	1	0.7
	Terminus segment	3	2.2
	Multiple localization	16	11.9
Vetebro–basilar artery	V4 segment of Vertebral Artery (*1 or multiple aneurysms*)	9	6.7
	P1 Segment of posterior cerebral artery	1	0.7
Total		134	100

### 3.2 Technical features

143 stents were used for 130 patients, among them 121 cases were treated with single stents, 9 cases needed 2 stents.4 cases were treated with combined stent and coil.

Pipeline was mostly used with 121 stent, accounted for 84.6%. FRED was applied to 21 stent, accounted for 14.7%. Silk stent, a new type in the market, was deployed in only one case. Average stent diameter was 4.3 mm (±0.38), and the mean stent length was 21.6 mm (±4.6).

Technical success rate was 96.3% (129 procedures), with good result (fully open in good position) of 85.8% (Table 2). 7 stents were not fully opened which was needed to ballooning. 2 cases had distal stent displacement, and this was lower than expected but still covered the neck of the aneurysms, 5 cases existed endoleak due to the wall of the parent arteries is not smooth (3.7%). Failure rates accounted for 3.7% due to not-opened-stents, displacement of the stent into the aneurysm or obstruction of the stent.

**Table 2:** Technical features.

Feature		N	%	Total
Success	Stent fully open in good position	115	85.8	n = 129 % = 96.3
	Stent not fully open (>75% diameter of the parent artery)	7	5.2	
	Displacement but still covered the neck of the aneurysms	2	1.5	
	Endoleak	5	3.7	
Unsuccessful	Stent not open	1	0.7	n = 5 % = 3.7
	Displacement of the stent into the aneurysm	2	1.5	
	Obstruction of the stent	2	1.5	

The majority of aneurysms immediately after stenting had a filling grade of ‘A’ occupying 83.5 % (Table 3). Cases of complete occlusion of the aneurysm right after intervention were due to coil and stent placement.

**Table 3:** Degree of angiographic filling of the aneurysms immediately after stenting (The O’KM scale classification).

Filling grade	N	%
A: Total filling (>95%)	111	83.5
B: Subtotal filling (5–95%)	15	11.3
C: Entry remnant (<5%)	5	3.8
D: No filling (0%)	2	1.5
Total	133	100.0

### 3.3 Treatment results

In 134 cases, 67 cases were followed-up for 3 months, and 60 cases were followed-up for 6–12 months (17 new cases that were not checked). The remaining cases lost connection. Total occlusion rates were 82.1% or 95% after 3 or 6–12 months, respectively according to Roy – Raymond’ Classification (Table 4).

**Table 4:** Results of aneurysms obstruction after intervention 3 months and 6–12 months (Roy – Raymond's Classification).

Filling grade	After 3 months		After 6–12 months	
	N	%	N	%
A: Total filling (>95%)	55	82.1	57	95
B: Subtotal filling (5–95%)	12	17.9	3	5
C: Entry remnant (<5%)	0	0	0	0
Total	67	100%	60	100

**Figure 2:**
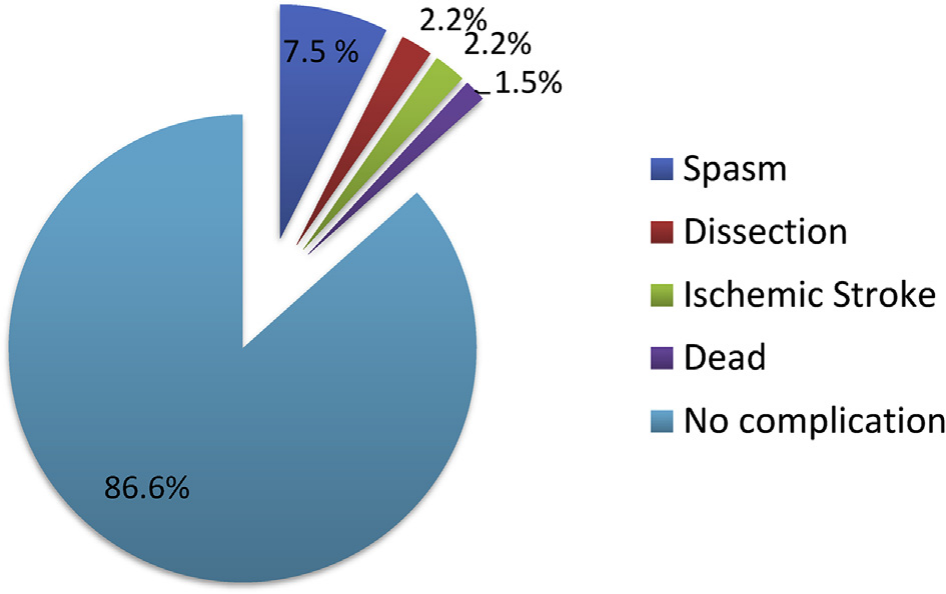
Mild and severe complications.

The incidence of severe complications accounted for 8/134 cases (6%), including dissection: 3 cases, cerebral infarction: 3 cases, and death: 2 cases (Subarachnoid hemorrhage grade 4 according to Fisher's Classification after 2 weeks and due to aneurysm rupture, stent obstruction) (Figure 2).

## 4 Discussion

Our study groups included 130 patients with a female/male ratio of 3.19 and an average age was 50 which was consistent with other previous studies [8, 9]. In this research, we found that most common locations of aneurysms were the cavernous and para-ophthalmic segments, otherwise multiple aneurysms at different segment of internal carotid artery, accounting for 35.1%, 40.3% %, and 11.9%. On the other hand, there was quite rare aneurysm located in the lower segments of internal carotid artery. It could be explained thanks to the thicker wall of petrouss bone segment. In many cases, the aneurysms have some branches arising from the wall, so it is quite difficult to preserve these branches with coiling. The wall of stents is mesh designed to ensure not to slow down the flow of these branches [10, 11]. In addition, our aneurysm diameter and height results are similar to previous studies [9, 12, 13]. Indication of flow diverter stent normally is very small or large and giant aneurysm, but recently, FDA expanded indication for Pipeline Flex with small and medium wide – neck aneurysm based on data result of PREMIER trial [14].

The incidence of wide-neck aneurysms <10 mm, >10 mm, and >20 mm, was 64.2%, 9% and 9%, respectively, and these aneurysms had high rate of revascularization as well as coil migration. Small aneurysms (<3 mm in diameter) or multiple aneurysms on a parent artery were also inconvenient for coiling. In five patients with blister-like aneurysms, one patient had subarachnoid hemorrhage treated throughout acute phase, was successfully reconstructive with stent and coil. Although blister-like aneurysms have small size (<3 mm), but they have very fragile wall due to dissection. In contrast, the giant one can hardly be completely packing by coil, but the combination of coil and stent can make progress blood clotting intra-aneurysmal and reduces the risk of displacement of stent into sac. Then the flow diverter were chosen in these case which are difficult or impossible to coiling [15].

We had chosen the stent depending on the diameter of the proximal landing zone expected of the damaged arteries, sized on three dimensional angiogram. The average diameter of the stent used was 4.3 mm (±0.38), and mean stent length was 21.6 mm (±4.6). If the stent is too large for the vessel, it will not open like design leading to thrombus formation earlier in the aneurysm. If the stent is smaller than the diameter of the vessel, there is a risk of leaking or displacement.

The technical success rate was 96.3% (129 cases) this result was consistent with other studies [9]. The stent not fully opened in 7 patients (5.2%) then needed to ballooning with good results.

The cases of stent mildly migrated but still completely covered the aneurysm neck or dissection of the parent artery without symptoms did not need to do more but needed to follow up the progressing. In one case of a fusiform aneurysm of V4 vertebral artery, shortened stent resulted in the head of the stent comind down into the aneurysm, needed to place 2th supplementary stent, follow up shown good results. Migration and shortening of the device can occur both in early and delay stage with fatal complication and deployment of additional stent is necessary if the neck of the aneurysm is not covered [16]. Navigation the giant aneurysm was very difficult because of turbulent flow; some solutions had been applied as a wire-loop technique, used solitaire stent or snared to fix the top of micro catheter [11].

**Figure 3:**
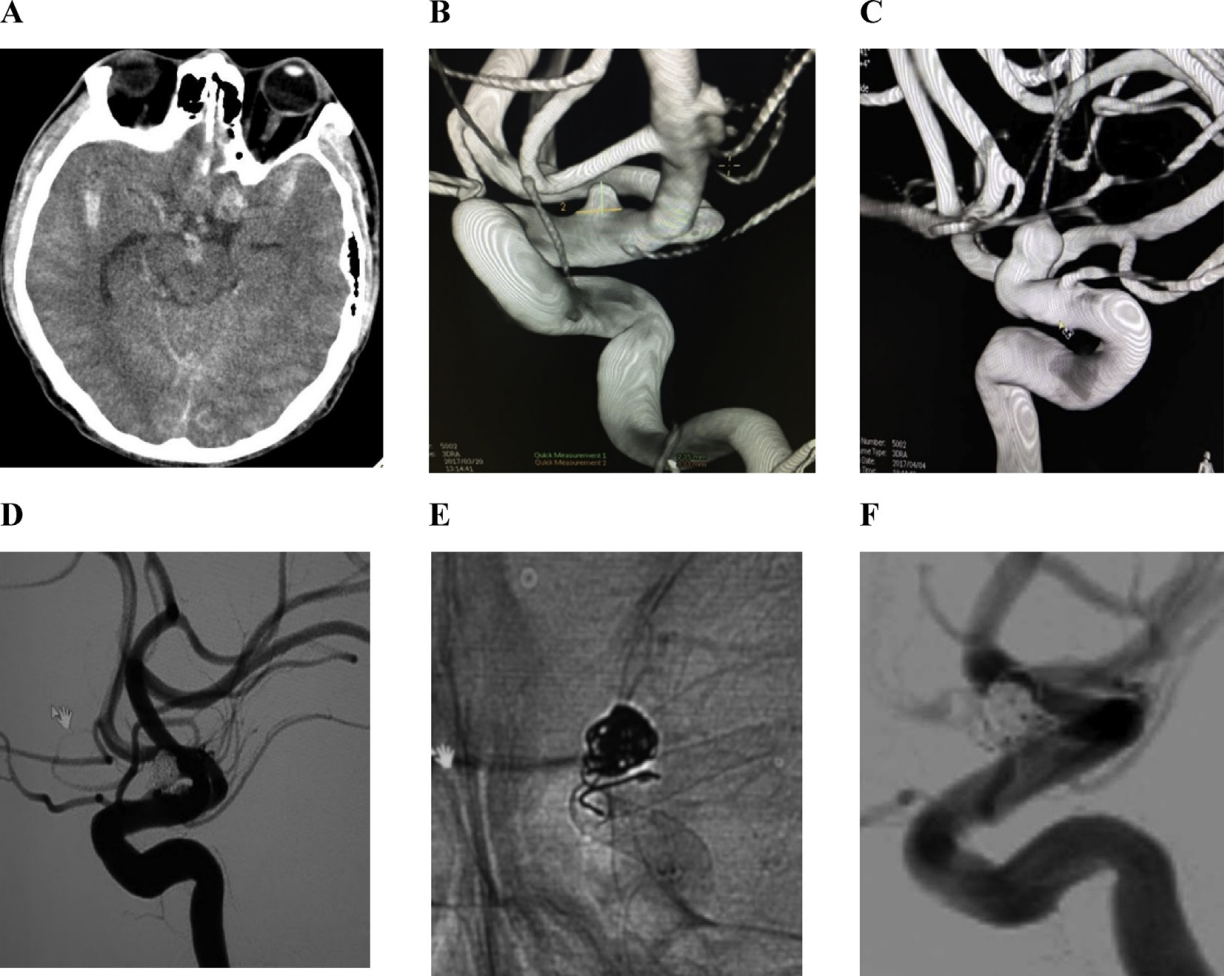
Flow diverted stent for ruptured blister like aneurysm. Male patient, 49 years old, admitted to hospital with headache, Glasgow coma scale (GSC): 15. CT-scan images showed subarachnoid hemorrhage of two hemispheres (A), DSA image showed a blister-like aneurysm of anterior choroidal segment of the right internal carotid artery (B), and bigger after 1 week (C). Good results with entry remnant filling (grade C) after coiling and deploying 1 Pipeline stent (D and E). DSA image control after 1 year showed total occlusion of the aneurysm (F).

In five unsuccessful cases that accounted for 3.7%: stent could not open in 1 case, stent migrated into the aneurysm sac in 2 cases and occlusion thrombose of stent in 2 cases of giant aneurysms leaded to dead or major stroke. In our study, a giant aneurysm of one patient ruptured into cavernous sinus after placement of the stent. After blocked by a detachable silicone balloon occlusion, the stent was dilated and angiogram showed good circulation flow, however, there was widespread left hemisphere ischemic stroke with hemorrhagic transformation leading to death. This was the case where the shortened stent was tipped into the aneurysm leading to hemodynamic disturbances and rupture and long time of intervention (nearly 3 h) lead to the formation of clots intra-stent. Another giant sac ruptured after deployment of the device without coiling in one patient at the 13^th^ day leading to death. In high risk of rupture or ruptured aneurysms like large, giant or blister-like one (Figure 3), putting some coils inside the sac additionaly to the flow-diverter is necessary to promote the thrombus proccess faster and reduce ruptured risk than using stent alone [17, 18]. In this series, we combine coil and stent in 4 cases with good result. The strategy and effectiveness of combining coil and flow diverter need more data to confirm. Right after deploying the stent, A1, A2, and A3 grades of angiographic filling according to the OKM scale were seen in majority with 111 cases, accounting for 83.5%, highly in the aneurysm less than 10 mm. In contrast, the giant aneurysms had grade higher according to thrombosis [7]. In two cases, the aneurysms obstruction was seen immediately after procedure due to coil and stent combination. We applied the OKM classification to compare the dynamics of flow in the aneurysm before and right after the stenting, but it did not replace the Roy-Raymond classification [19].

CTAngio and MRAngio of 67 cases were recorded after 3 months follow up. The rate of aneurysm completed occlusion, stent patency and normal cerebral parenchyma accounted for 82.1%, consistent with Lubicz's study [20], and increased to 95% after 6–12 months. The rate of aneurysm uncompleted occlusion, stent patency and normal cerebral parenchyma accounted for 17.9%, seen commonly in large aneurysms >10 mm, but the overall size of the aneurysms decreased. Small one (<10 mm) tend to disappear rather than thrombosis, as observed on images. CT-Scan had higher resolution than MRI in evaluating stent patency, stenosis, as well as better observation to size the remnant part. In fact, MRI is superior in the diagnosis of cerebral infarction. Some authors suggest MRI 3T is promising tool to follow up intracranial aneurysms treated by flow diversion [21, 22].

## 5 Conclusion

Aneurysms accounted for the high incidence in the carotid artery at the cavernous and anterior choroid segments. The most common types were wide-neck and multiple sacs in one parent artery. Application of the flow diverter stent was a safe and effective treatment with high success rate (96.3%) and low complication (3.7%). Imaging follow-up was important to evaluate the state of aneurysm, and parent vessel and cerebral parenchyma allowed to make decisions for the patient. CT-Scan showed better assessment of the aneurysm as well as the stent than MRI, but low sensitivity to small cerebral infarct lesions.

## Declarations

### Author contribution statement

D. T. Chu, M. J. Uddin, T. C. Dinh: conceived and designed the experiments; analyzed and interpreted the data; wrote the paper.

N. T. Binh: conceived and designed the experiments; performed the experiments; analyzed and interpreted the data; contributed reagents, materials, analysis tools or data; wrote the paper.

V. D. Luu, P. M. Thong, N. N. Cuong, N. Q. Anh, T. A. Tuan, L. T. Linh, N. T. Thien: conceived and designed the experiments; performed the experiments; contributed reagents, materials, analysis tools or data.

### Funding statement

This research did not receive any specific grant from funding agencies in the public, commercial, or not-for-profit sectors.

### Competing interest statement

The authors declare no conflict of interest.

### Additional information

No additional information is available for this paper.

## References

[1] Molyneux A.J. (2005). International subarachnoid aneurysm trial (ISAT) of neurosurgical clipping versus endovascular coiling in 2143 patients with ruptured intracranial aneurysms: a randomised comparison of effects on survival, dependency, seizures, rebleeding, subgroups, and aneurysm occlusion. Lancet.

[2] Steiner T. (2013). European stroke organization guidelines for the management of intracranial aneurysms and subarachnoid haemorrhage. Cerebrovasc. Dis..

[3] Hackenberg K.A.M., Hänggi D., Etminan N. (2018). Unruptured intracranial aneurysms. Stroke.

[4] Wermer M.J.H. (2007). Risk of rupture of unruptured intracranial aneurysms in relation to patient and aneurysm characteristics. An Updated Meta-Analysis.

[5] Lylyk P. (2009). Curative endovascular reconstruction of cerebral aneurysms with the pipeline embolization device: the Buenos Aires experience. Neurosurgery.

[6] Fiorella D. (2008). Definitive reconstruction of circumferential, fusiform intracranial aneurysms with the pipeline embolization device. Neurosurg. Clin..

[7] Fischer S. (2012). Pipeline embolization device (PED) for neurovascular reconstruction: initial experience in the treatment of 101 intracranial aneurysms and dissections. Neuroradiology.

[8] Berge J. (2012). Flow-diverter Silk stent for the treatment of intracranial aneurysms: 1-year follow-up in a multicenter study. Am. J. Neuroradiol..

[9] Nelson P.K. (2011). The pipeline embolization device for the intracranial treatment of aneurysms trial. Am. J. Neuroradiol..

[10] Kallmes D.F. (2007). A new endoluminal, flow-disrupting device for treatment of saccular aneurysms. Stroke.

[11] Kan Peter (2015). Techniques in distal access of wide-necked giant intracranial aneurysms during treatment with flow diversion. Surg. Neurol. Int..

[12] Pierot L. (2019). SAFE study (Safety and efficacy Analysis of FRED Embolic device in aneurysm treatment): 1-year clinical and anatomical results. J. Neurointerventional Surg..

[13] Pierot L. (2018). Feasibility, complications, morbidity, and mortality results at 6 months for aneurysm treatment with the Flow Re-Direction Endoluminal Device: report of SAFE study. J. Neurointerventional Surg..

[14] Hanel R.A. (2019). Prospective study on embolization of intracranial aneurysms with the pipeline device: the PREMIER study 1 year results. J. Neurointerventional Surg..

[15] Briganti F. (2015). Endovascular treatment of cerebral aneurysms using flow-diverter devices: a systematic review. NeuroRadiol. J..

[16] Chalouhi N. (2013). Spontaneous delayed migration/shortening of the pipeline embolization device: report of 5 cases. Am. J. Neuroradiol..

[17] Yang C., Vadasz A., Szikora I. (2017). Treatment of ruptured blood blister aneurysms using primary flow-diverter stenting with considerations for adjunctive coiling: a single-centre experience and literature review. Intervent Neuroradiol. : J. Peritherapeutic Neuroradiol. Surg. Proced. Relat. Neurosci..

[18] Brinjikji W. (2016). Treatment of ruptured complex and large/giant ruptured cerebral aneurysms by acute coiling followed by staged flow diversion. J. Neurosurg..

[19] Raymond J. (2003). Long-term angiographic recurrences after selective endovascular treatment of aneurysms with detachable coils. Stroke.

[20] Lubicz B. (2010). Flow-diverter stent for the endovascular treatment of intracranial aneurysms: a prospective study in 29 patients with 34 aneurysms. Strocke.

[21] Attali J. (2016). Follow-up of intracranial aneurysms treated by flow diverter: comparison of three-dimensional time-of-flight MR angiography (3D-TOF-MRA) and contrast-enhanced MR angiography (CE-MRA) sequences with digital subtraction angiography as the gold standard. J. Neurointerventional Surg..

[22] Guan J. (2017). High-resolution magnetic resonance imaging of intracranial aneurysms treated by flow diversion. Interdiscipl. Neurosurg..

